# Reading *Sky* and Seeing a Cloud: On the Relevance of Events for Perceptual Simulation

**DOI:** 10.1037/xlm0000318

**Published:** 2016-10-20

**Authors:** Markus Ostarek, Gabriella Vigliocco

**Affiliations:** 1 Experimental Psychology Department, Division of Psychology and Language Sciences, University College London, and Max Planck Institute for Psycholinguistics, Nijmegen, the Netherlands; 2 Experimental Psychology Department, Division of Psychology and Language Sciences, University College London

**Keywords:** embodied cognition, language comprehension, language–perception interactions

## Abstract

Previous research has shown that processing words with an up/down association (e.g., *bird*, *foot*) can influence the subsequent identification of visual targets in congruent location (at the top/bottom of the screen). However, as facilitation and interference were found under similar conditions, the nature of the underlying mechanisms remained unclear. We propose that word comprehension relies on the perceptual simulation of a prototypical event involving the entity denoted by a word in order to provide a general account of the different findings. In 3 experiments, participants had to discriminate between 2 target pictures appearing at the top or the bottom of the screen by pressing the left versus right button. Immediately before the targets appeared, they saw an up/down word belonging to the target’s event, an up/down word unrelated to the target, or a spatially neutral control word. Prime words belonging to target event facilitated identification of targets at a stimulus onset asynchrony (SOA) of 250 ms (Experiment 1), but only when presented in the vertical location where they are typically seen, indicating that targets were integrated in the simulations activated by the prime words. Moreover, at the same SOA, there was a robust facilitation effect for targets appearing in their typical location regardless of the prime type. However, when words were presented for 100 ms (Experiment 2) or 800 ms (Experiment 3), only a location nonspecific priming effect was found, suggesting that the visual system was not activated. Implications for theories of semantic processing are discussed.

Recent theories of semantic processing have identified two mechanisms that are likely involved in language comprehension. According to the first, word meaning is represented in a distributed network based on sensory experience. Comprehension, in this view, relies at least partly on simulations of sensory experience in one or several modality-specific areas (Barsalou, 1999, Pulvermüller, 2005), as well as their integration in convergence zones (Binder & Desai, 2011; Damasio, Tranel, Grabowski, Adolphs, & Damasio, 2004; Simmons & Barsalou, 2003). Although there is mounting evidence that conceptual information is distributed over modality-specific sensory and multimodal areas in the cortex (Barsalou, 1999, 2008; Hoffman & Lambon Ralph, 2013; Kiefer & Pulvermüller, 2012; Kiefer & Trumpp, 2012; Meteyard, Cuadrado, Bahrami, & Vigliocco, 2012; Pulvermüller, 1999, 2013), the emerging picture is that the involvement of sensory systems is flexible and dependent on contextual factors such as task requirements (e.g., Lebois, Wilson-Mendenhall, & Barsalou, 2015; Zwaan, 2014). As the field matures, the main questions have shifted to more fine-grained problems such as when and how simulation contributes to language comprehension.

The second mechanism is based on the idea that co-occurrence statistics between words can be used as a source to semantic information (dual coding theory [Paivio, 1990], 2013; lexical hypothesis [Glaser, 1992]; language and situated simulation theory [Barsalou, Santos, Kyle, & Wilson, 2008; Louwerse, 2011]; integrated systems [Andrews, Vigliocco, & Vinson, 2009; Vigliocco, Meteyard, Andrews, & Kousta, 2009]). Models based on this account have proved to be very successful at predicting human behavior in a surprising array of tasks (e.g., Landauer & Dumais, 1997). Because of its focus on internal distributional patterns, however, it has been proposed that it needs to be combined with the experience-based account (Andrews et al., 2009) in order to benefit from grounded representations that can directly interface with the world. In the current study, we provide evidence pertaining mainly to the question of when perceptual simulation occurs, but also to the question of how the relative role of simulation and distributional patterns unfolds over time during online word comprehension.

One promising line of research for tackling such questions has been the study of words denoting objects with a certain spatial association on (e.g., Estes, Verges, & Barsalou, 2008)—as well as words and sentences describing motion along (Meteyard, Bahrami, & Vigliocco, 2007; Meteyard, Zokaei, Bahrami, & Vigliocco, 2008)—the vertical axis. Most studies used variations of an experimental paradigm in which the participants have to perform a simple visual (discrimination or detection) task with meaningless targets, for example, X versus O, appearing at the top or bottom of the screen. Crucially, before each trial, they see task-irrelevant words referring to objects that are usually encountered in the upper (e.g., *bird*) or lower (e.g., *mouse*) visual field. Although various studies have shown that spatial associations of words can influence visual perception in such tasks, it is still debated to what extent such effects are automatic and to what extent they require simulations.

A key (and, at first sight, puzzling) feature of the available literature is that words with up/down associations can both interfere with (Bergen, Lindsay, Matlock, & Narayanan, 2007; Dudschig, Lachmair, de la Vega, De Filippis, & Kaup, 2012; Estes et al., 2008; Gozli, Chasteen, & Pratt, 2013; Verges & Duffy, 2009), or facilitate the discrimination of (Chasteen, Burdzy, & Pratt, 2010; Gozli et al., 2013; Zhang et al., 2013), visual targets in congruent locations. Gozli and colleagues (2013) scrutinized this pattern of findings and showed that interference in compatible location occurs only when SOA is short (<400 ms), whereas facilitation is observed at longer SOAs. They identified one exception to this rule, however—namely, that facilitation is observed also at short SOAs when all cue words are from the same semantic field (religion-related and house-related words; Gozli et al., 2013, Experiments 5 and 6).

Here, we introduce the notion of event simulation to account for this set of findings: Following the logic of embodied semantics, we hypothesize that reading up/down words referring to objects (such as *bird*) elicits simulations of the actual perception of these objects in visual brain areas. Crucially, we do not usually see things in isolation, but together with other things creating a visual event (“situated conceptualization”; Barsalou, 2009). Many entities tend to co-occur with specific entities (e.g., the sky and clouds) in a prototypical event. If conceptual processing is based on perceptual experience, words should activate simulations not only of the entities they denote but also of the whole event in which the entity is typically embedded, including frequently co-occurring entities. This can account for the findings sketched in the last two paragraphs: If, at the same time that a visual event is simulated in the upper visual field (e.g., a bird flying in the sky) an unrelated object has to be identified in the same location (e.g., a X), interference should result because the simulation process uses resources that would be required for the visual task (explaining early interference). After the simulation is accomplished, attention to upward location may remain at an increased level (explaining late facilitation). Event simulation can also explain why interference occurs only when cue words are from multiple semantic categories: When cues from only one single semantic category are used (such as house-related words), they all tap the same background event and thereby augment its activation level. Consequently, after some trials, reactivating the relevant event is much less effortful, resulting in less competition between visual processing resources required for event simulation and those required for the visual discrimination task.

In line with this, Estes, Verges, and Adelman (2015) recently found (in Experiments 1 and 2) faster responses to pictures in the cued location when the cue word was the label of the respective picture (e.g., “bird” → picture of a bird), whereas the same word followed by an unrelated picture (e.g., of a wrench) led to slower responses in the cued location (the top of the screen). Though highly relevant, these experiments suffer of a number of limitations. First, although the authors describe the matching targets as semantically related, they also closely match the perceptual expectations triggered by the prime word, which are hypothesized to be the driving force behind the perceptual matching account. It therefore remains unclear whether semantically matching, but perceptually mismatching, prime-target pairs yield facilitation. Second, pictures in the related condition themselves had a typical location they are strongly associated with (a bird is typically up in the sky), whereas the pictures in the unrelated condition did not. It is thus unclear whether the reason that a picture of a bird was responded to faster at the top of the screen after the prime “bird” was that it was semantically primed, that it matched the expected shape, or that it appeared in its typical location. Our study takes these factors into account by using targets with typical locations across all conditions and by using typically co-occurring but perceptually dissimilar prime-target pairs to probe the activation of location-specific event simulations. Finally, Estes and colleagues (2015) focused on one particular effect—namely, the interference effect at a SOA of 150 ms, while one of the key findings is that the effect of spatial words on perception changes dynamically over time. In three experiments below, we systematically manipulated SOA to track the dynamic activation of visuospatial features during word comprehension.

## The Present Study

To test the hypothesis that word comprehension involves the automatic activation of events as described in the Introduction, the previously used paradigm was adapted in the following way: Instead of meaningless symbols (such as X/O), the targets were drawings of objects that either did or did not belong to the prime word’s prototypical event (e.g., *sky* → picture of a cloud). Our main prediction, based on the notion of event simulation, is that prime-target pairs belonging to the same prototypical event should yield facilitation in the cued location at a short SOA, whereas event-unrelated prime-target pairs should lead to slower responses in the cued location, based on previous studies (e.g., Estes et al., 2008). This is because perception of targets should benefit from the event that is activated by event-related, but not event-unrelated, words. We thus predict a location-specific priming effect, as priming by event-related words is only expected when the target appears in the location that is compatible with the respective real-world event the word activates.

## Experiment 1

In Experiment 1, we used an SOA of 250 ms, which typically results in interference when meaningless symbols are used as targets (Estes et al., 2008; Gozli et al., 2013). We predicted that interference would be replaced by facilitation when the target picture and prime word belong to the same event; thus, after reading *sky*, a picture of a cloud should be identified faster at the top, compared with the bottom, of the screen. We further predicted that this effect should be reversed for prime-target pairs that are not event-related, based on previous studies (e.g., Estes et al., 2008).

### Method

#### Participants

Twenty-four subjects participated for payment. All participants had normal or corrected-to-normal vision and were native English speakers. One participant was excluded because of vision problems, two were excluded because of very slow responses (more than 2.5 *SD*s from the mean), and one participant was excluded because she reported knowing about the aim of the study. The data from the remaining 20 subjects were used for all analyses (one left-handed, 10 female, age = 21–74 years, *M* = 28.05, *SD* = 11.97). None of the participants suspected the spatial associations of the prime words or the vertical axis to be central to the experiment. The most common observation was that some of the words were associated with each other.

#### Materials

Eight black-and-white drawings of objects typically occurring in an upper location (e.g., a cloud) and eight drawings of objects typically occurring in a lower location (e.g., a shoe) were selected from Snodgrass and Vanderwart (1980) or had nearly identical characteristics (*n* = 3). Ten volunteers rated their typical location on a 5-point Likert scale (1 = *up*; 5 = *down*), confirming that the up and down target pictures were associated with upper and lower space, respectively (up-targets = 1.7; down-targets = 4.6; *p* < .001). The pictures were shrunk so that they had a size of approximately 4 cm × 3 cm on the computer screen (PC: Dell Optiplex 960; monitor: Dell P190S; screen size = 19 in.; resolution = 1280 × 1024; refresh rate = 60 Hz). In each of the eight experimental blocks, one picture of each category (up and down) served as a target that was responded to by pressing the left or right button on a button box. Prime words belonged to three categories: The first category included 16 words denoting entities that typically co-occur with one of the targets (e.g., *sky* → cloud). To be more precise, these prime words referred to entities that belong to the prototypical event associated with the target; for instance, the event of looking in the sky typically involves seeing clouds. All words from this category also shared the spatial association with the targets they were related to. The second category included 16 words having the same spatial association as one of the targets, but not typically co-occurring with them (e.g., *root* → shoe). The third category included 32 neutral control words without a vertical association (*skin*, *beer*, etc.). The three categories were matched for frequency (based on the SUBTLEX-UK database; Van Heuven, Mandera, Keuleers, & Brysbaert, 2014), number of syllables, and length (all *p* values > 0.4). Ten volunteers rated the spatial associations of all stimuli on a 5-point Likert scale (1 = *up*; 5 = *down*), validating the primes’ a priori determined membership in the three categories (see Table 1). Additionally, they confirmed that the prime-target relatedness is specific to event-related prime-target pairs (see Table 2). To further rule out the possibility that there were confounds between conditions in terms of semantic similarity, we retrieved latent semantic analysis cosines for each prime-target pair (Landauer, Foltz, & Laham, 1998). Cosine values in the event-related condition were much higher than in the spatially congruent, *t*(15) = 6.678, *p* < .001, and the control, *t*(15) = 6.589, *p* < .001, conditions, whereas there was no difference between the spatially congruent and control conditions (*t* < 1).[Table-anchor tbl1][Table-anchor tbl2]

Prime words were presented in black (14-point Arial font) against a white background. E-Prime (Psychology Software Tools, Inc., Pittsburgh, PA; www.pstnet.com) was used for stimuli presentation and data collection.

#### Procedure

**Figure 1 fig1:**
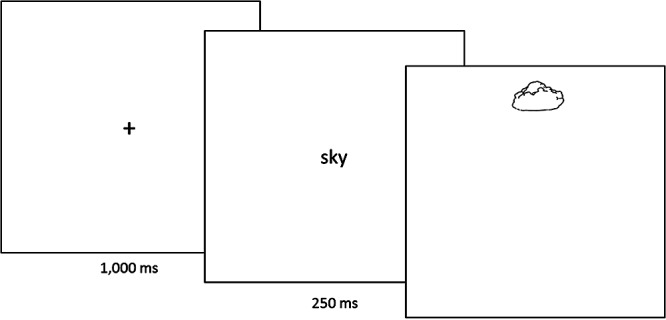
Depiction of an event-related and spatially congruent trial.

The subjects were seated in a sound-attenuated room and were presented with eight experimental blocks^1^ of approximately 4 min, resulting in an overall duration of approximately 35 min. During the whole experiment, the participants kept their chin on a chin rest 52 cm from the computer screen. At the beginning of each trial, a fixation cross appeared for 1,000 ms, followed by a prime word in central position presented for 250 ms. Then, one of the target pictures appeared at the top (center of the picture 9° above the center), bottom (9° below the center), or in central position until a response was made (see Figure 1).[Fig-anchor fig1]

Each block had two target pictures. Subjects were told to press the left button for one of the pictures and the right button for the other one as fast and as accurately as possible (left/right responses were counterbalanced within and across participants). Per block there were eight different prime words: two denoting objects typically co-occurring with one of the targets, two spatial (up and down) words that were not related to the targets, and four spatially neutral control words. Prime words were repeated six times, such that they were paired once with each of the two targets in upper, lower, and central location, resulting in 48 trials per block. The order of the prime words in each block as well as the order of the blocks was pseudorandomized. Subjects were told that they had to pay attention to the words and after each block they had to do a memory test: They were presented with three words and had to decide whether they saw them during the last block or not. The words in the memory test were selected randomly from a list containing the eight prime words that occurred during the block and another eight words that did not appear as prime words. This was to ensure that the participants attended to the prime words. To make sure that they were familiar with the pictures and the corresponding buttons they had to press, there were 24 practice trials before each block. In the practice trials, the prime words were replaced by the color words *red*, *blue*, *green*, and *yellow*, and feedback appeared on the screen (“Correct” or “Incorrect”) for 500 ms following each response. In the test phase, the subsequent trial started immediately after each response without feedback.

#### Design and analyses

The experiment was mainly designed to investigate the effect of words with a vertical association on the discrimination of visual targets. As in previous studies, one key manipulation was whether the target appeared in the cued versus uncued location (spatially congruent vs. incongruent). Additionally, targets either belonged to the prime’s prototypical event or were event-unrelated. This resulted in a 2 × 2 within-subjects design that we analyzed using a repeated measures ANOVA with the factors Event Congruence (event-related vs. event-unrelated) and Spatial Congruence (spatially congruent vs. spatially incongruent). All primes were paired with the targets in upper, lower, or central location. Targets in central location only had the function to prevent the subjects from establishing a polarity correspondence (PPC; Cho & Proctor, 2003; Proctor & Cho, 2006) between binary features of stimuli (up vs. down) and response code (left vs. right) and were not included in the analyses.^2^ We also included a control condition with spatially and event-unrelated primes. This condition was not included in the ANOVA we report.

One final factor of interest was analyzed separately from the main analysis: As all targets were pictures of objects that themselves typically occur in upper or lower location, their location on the screen was either compatible or incompatible with their typical location, which could affect response times.^3^ This was tested with a paired-samples *t* test.

### Results and Discussion

Of main interest was whether words that typically co-occur with one of the target pictures induce faster responses when the target appears in the cued location (*sky* → cloud-up) versus the opposite location (*sky* → cloud-down). Inspection of the results in each individual block indeed revealed a consistent advantage in this condition, with one exception: One block showed the opposite pattern. In this block, one of the critical prime-picture pairs was *blossom* → flower, which was supposed to have a down association. However, it is problematic that the blossom is the upmost part of a flower and therefore may be considered an “up word.” Its spatial association rating of 2.7 (1 = up; 5 = down) confirms this suspicion. Note also that this prime word has previously been used as an “up word” in Estes et al. (2008). For these reasons, the block containing flower as a target was not included in the analyses.

#### Memory scores

Overall, participants were 91% accurate in the forced choice memory test after each block (range = 79%–100%), indicating that they were indeed reading the words and not just responding to the targets.

#### Reaction time (RT) analysis

**Figure 2 fig2:**
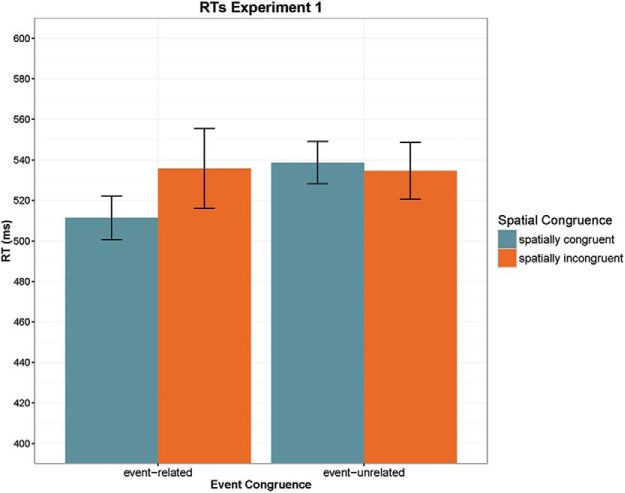
Summary of reaction times (RTs) in Experiment 1 plotted as a function of event congruence and spatial congruence. Error bars indicate 95% confidence intervals. See the online article for the color version of this figure.

The 2 × 2 ANOVA revealed a marginally significant main effect of Event Congruence, *F*(1, 19) = 3.753, *p* = .068, η_p_^2^ = 0.165, with shorter RTs in the event-related (*M* = 523 ms, *SE* = 18 ms) versus event-unrelated condition (536 ms, *SE* = 17 ms). Crucially, there was a significant interaction between Event Congruence and Spatial Congruence, *F*(1, 19) = 4.723, *p* = .043, η_p_^2^ = 0.199, reflecting faster responses to targets in the cued location when the prime was event-related to the target and slightly slower responses to targets in the cued location when the prime was not event-related to the target (see Figure 2). A follow-up paired-samples *t* test confirmed that, as predicted, priming in the event-related condition was location specific: *sky* → cloud-up was faster (*M* = 511 ms, *SE* = 19 ms) than *sky* → cloud-down (*M* = 535 ms, *SE* = 21 ms), *t*(19) = −2.162, *p* = .044. However, responses to event-unrelated targets in the cued location were not significantly slowed down (cued: *M* = 538 ms, *SE* = 17 ms; uncued: *M* = 534 ms, *SE* = 18 ms, *t* < 1). We thus did not replicate the interference effect in the cued location after semantically unrelated primes observed by Estes et al. (2008, 2015). To test for the possibility that the big number of target repetitions might have masked potential effects, we coded the data for first versus second half for each experimental block and reran the ANOVA including this factor as additional factor. It did not interact with any of the factors of interest (*p* > .2).[Fig-anchor fig2]

Finally, to test whether there was an overall advantage for targets in their typical location, we compared averaged RTs from all conditions with targets in typical versus atypical location. A paired-samples *t* test revealed a robust facilitation effect for targets in typical location (mean difference: 18 ms), *t*(19) = −4.325, *p* < .001.

#### Error analysis

The same analyses were performed on accuracy rates. The 2 × 2 ANOVA showed no significant effects.

### Summary

The results confirm the predicted location-specific priming effect in the event congruent condition but not in the event incongruent condition. This suggests that word comprehension involves simulation not only of the denoted entities, but of visual events they are typically embedded in. The scenario we propose is that reading *sky* triggers the partial reenactment of an upward gaze into the sky, where clouds are typically seen. Therefore, trials in the event-related condition were either compatible or incompatible with the event simulation (depending on spatial congruence): After reading *sky*, the detection of a cloud at the top of the screen is facilitated, whereas there is a disadvantage for a cloud appearing at the bottom. Interestingly, we observed virtually no effect of spatial congruence in the event-unrelated condition and, particularly, we found no evidence for interference in the cued location. This is at odds with several previous studies (e.g., Estes et al., 2008, 2015; Gozli et al., 2013). One possible explanation for this difference is the overall advantage for targets in typical location, which may have overridden the interference effect. Inspection of the effect of individual prime types showed that all conditions with words having a spatial association contributed to the effect (even those cuing the opposite location), and that only the control condition with spatially neutral words did not. Our interpretation of this result is that spatial words activate the visuospatial system, which subsequently is more sensitive to the targets’ locations on the screen and to the compatibility with their typical locations in the real world. Spatially neutral words, on the other hand, do not preactivate it, and typical versus atypical target location thus does not modulate RTs. This is reminiscent of studies that have shown faster RTs to words in the typical location of their referents (*branch* at the top, *snake* at the bottom) even without prime words (Šetić & Domijan, 2007; Zwaan & Yaxley, 2003).

## Experiment 2

Experiment 1 indicates that embodied effects arise early on during semantic processing. It also indicates that they occur rather automatically, because no attention was explicitly drawn to the vertical axis and none of the participants suspected that it was relevant for the study. The following experiment addresses the question of what happens at an even earlier stage of processing by reducing the SOA to 100 ms. Strong versions of embodied cognition hold that simulation is necessary and sufficient for conceptual processing and predict that sensory information is retrieved at the earliest stages of semantic processing. According to this account, we should replicate the results from Experiment 1. Alternatively, there could be an initial stage of shallower lexicosemantic processing based on word co-occurrence statistics before richer sensorimotor representations are activated, which would predict no location-specific effects but significant effects of event congruency.

### Method

#### Participants

Twenty-four subjects participated in Experiment 2. Four subjects were excluded (a) because of tiredness and very slow responses, (b) because of impaired vision, (c) because they did not fulfil the native speaker requirement, and (d) because of low accuracy in the memory test (38%). The data from the remaining 20 participants were used for all analyses (one left-handed, 10 female, age = 19–34 years, *M* = 23.0, *SD* = 3.48). Again, none of the participants reported having suspected the vertical axis to be of importance for the experiment, while nearly all subjects spotted the occasional semantic prime-target associations.

#### Materials

Materials were identical with those of Experiment 1, with the exception that the block containing *blossom* → flower) was removed, resulting in seven instead of eight blocks.

#### Procedure

The procedure was the same as in Experiment 1, with the exception that prime words were presented for 100 ms instead of 250 ms.

### Results and Discussion

#### Memory scores

Overall, participants were 82.2% accurate in the memory test (range = 57%–100%), indicating that the reduced duration of prime presentation made it slightly harder to read the words.

#### RTs

**Figure 3 fig3:**
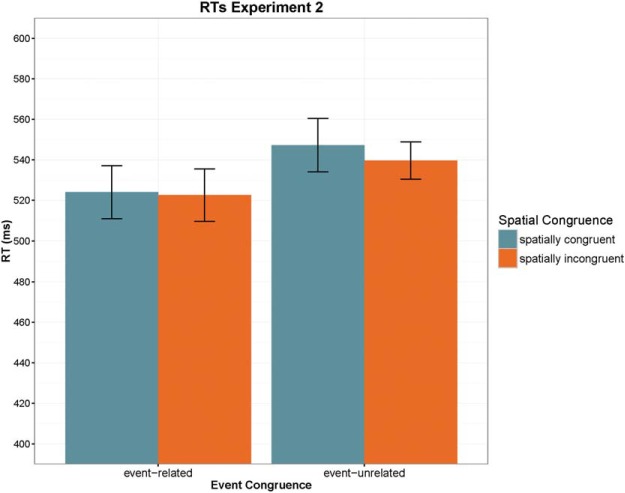
Summary of reaction times (RTs) in Experiment 2 plotted as a function of event congruence and spatial congruence. Error bars indicate 95% confidence intervals. See the online article for the color version of this figure.

The 2 × 2 ANOVA revealed only a significant effect of Event Congruence reflecting faster responses in the event-related condition, *F*(1, 19) = 17.734, *p* < .001, η_p_^2^ = 0.483, whereas neither Spatial Congruence nor the interaction was significant (see Figure 3). In contrast to Experiment 1, the advantage after event-related primes was completely location nonspecific, as shown by a paired samples *t* test comparing RTs in the event-related condition with spatially congruent versus incongruent targets (*p* > .8). It is thus a location nonspecific semantic priming effect. Again, we found no evidence for slower responses in the cued location after event-unrelated words (7.6 ms, *t* < 1). Finally, the separate *t* test comparing typical versus atypical target location showed no effect (mean difference = 2 ms, *t* < 1).[Fig-anchor fig3]

#### Error analysis

The error analysis revealed no significant effects.

### Summary

Our data suggest that at this early stage of processing, the visuospatial system is not activated. The strongest possible account of embodied word processing would argue that no conceptual processing can happen without sensorimotor contributions. However, the main effect of Target Location and the location-specific priming effect from Experiment 1, which we interpret as indices of event simulation, have completely vanished. At the same time, a location nonspecific priming effect has been found, indicating that some semantic processing is taking place. This is consistent with many studies on associative priming at short SOAs (see Neely, 1991, for a review). It is possible that at this early point in time, semantic processing is shallow and participants rely on statistical knowledge about co-occurrences of words (Louwerse, 2008; Louwerse & Jeuniaux, 2010). The idea that a linguistic system can stand aside a modal system in word processing has been repeatedly proposed (dual coding theory [Paivio, 1990, 2013]; lexical hypothesis [Glaser, 1992]; language and situated simulation theory [Barsalou et al., 2008; Louwerse, 2011]; integrated systems [Andrews et al., 2009; Vigliocco et al., 2009]) and recently received support from behavioral studies (Hargreaves & Pexman, 2014; Louwerse & Jeuniaux, 2010). Our results are consistent with the idea that although this mechanism taps into the relations between words, it does not provide in-depth featural information about the given concepts (such as where its referent is typically found). Such information seems to be provided slightly later during word processing via simulation.

## Experiment 3

Experiments 1 and 2 suggest that perceptual simulations are activated around 250-ms postword onset, a time window in which semantic processing is likely to take place. An open question is whether the observed effects reflect necessary semantic processes or rather optional conceptual “coloring” in the form of activation spreading from disembodied representations to sensory areas (Mahon & Caramazza, 2008). On the latter account, the role of perceptual simulations is to optionally enrich conceptual processing by means of mental imagery after core conceptual information is activated. Consequently, simulation-style effects are likely to be strongest when there is enough time for rich, imagery mediated, conceptual activation. The latter proposal therefore predicts stronger embodied effects at longer SOAs, a prediction that will be tested by increasing the SOA to 800 ms. The former proposal, by contrast, holds that simulation constitutes the comprehension process itself. Based on converging results from numerous electroencephalography studies, it is well established that word comprehension is typically accomplished within 400 ms after word onset. Therefore, the embodied semantics account predicts that simulation effects should peak in this early processing window and rapidly decay thereafter. Our choice of an SOA of 800 ms is based on several previous studies using similar implicit spatial cuing paradigms which found facilitation effects at 800-ms SOAs, consistent with a late imagery explanation (e.g., Chasteen et al., 2010; Gozli et al., 2013). It is, however, important to also note here that in these studies, a categorization task had to be performed on the prime words before the visual target appeared, such that processes beyond what is necessary for comprehension were likely involved. Here, we tap into the automatic processes engaged in word comprehension by avoiding the additional semantic categorization task.

### Method

#### Participants

Twenty-three subjects participated in Experiment 3. Two had to be excluded because of technical failure and one because of extremely slow responses. The data from the remaining 20 participants were used for all analyses (two left-handed, 14 female, age = 19–52 years, *M* = 25.4, *SD* = 9.11). Again, none of the participants reported having suspected the vertical axis to be of importance for the experiment, and nearly all subjects spotted the prime-target associations.

#### Material and procedure

Material and procedure were identical to Experiment 1, with one exception: prime duration was 800 ms.

### Results and Discussion

#### Memory scores

Overall, participants were 96% accurate in the memory test (range = 81%–100%), indicating that the longer prime presentation made it easier to memorize the words.

#### RTs

**Figure 4 fig4:**
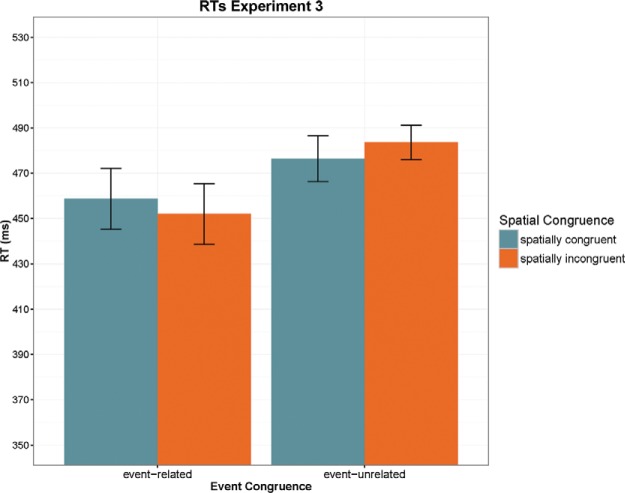
Summary of reaction times (RTs) in Experiment 3 plotted as a function of event congruence and spatial congruence. Error bars indicate 95% confidence intervals. See the online article for the color version of this figure.

The 2 × 2 ANOVA showed a significant main effect of Event Congruence with faster responses in the event-related versus unrelated condition, *F*(1, 19) = 22.8, *p* < .001, η_p_^2^ = 0.545. The main effect of Spatial Congruence and the interaction were both nonsignificant (see Figure 4). Again, priming for event-related prime-target pairs was not location-specific (*t*-test event-related condition with target in congruent vs. incongruent location, *ns*, *p* > .5), there was no interference in the cued location for event-unrelated targets (*t* < 1), and no facilitation for targets in their typical location (*t* < 1).[Fig-anchor fig4]

#### Error analysis

The error analysis showed no significant effects.

### Summary

Thus, as in Experiment 2, there was an associative priming effect but no location-specific effects. Previous studies using a similar paradigm and a SOA of 800 ms have found a congruency effect (see Chasteen et al., 2010, and Gozli et al., 2013, for a replication). A possible explanation for this difference may be that in those studies, the cue words were abstract words and had to be categorized before the visual discrimination task was performed. Therefore, participants probably processed the stimuli in more depth than in the present experiment, leading to longer-lasting perceptual simulations. This suggests that late congruency effects reflect processes which are not necessary for word comprehension and can be absent without an artificial semantic task. Experiment 1 by contrast, is likely to reflect core processes of word processing.

## Analysis of Experiments 1–3

Our interpretation of the data is that the location-specific priming effect and the shorter responses to targets in their typical location were specific to Experiment 1 (SOA = 250 ms). In order to confirm the impression that location-dependent priming in the event-related condition was specific to the SOA of 250 ms, we conducted a follow-up ANOVA including Congruency (event-related and target in cued location vs. event-related and target in uncued location) as a within-subjects factor and SOA (250 ms vs. 100 ms and 800 ms) as a between-subjects factor. We indeed found a significant interaction between SOA and Congruency, *F*(2, 58) = 5.173, *p* = .027, η_p_^2^ = 0.082. Planned *t* tests showed that event-related targets were responded to significantly faster in cued versus uncued location at SOA 250 ms, *t*(19) = −2.162, *p* = .044, whereas there was no significant difference at SOA 100 ms (*p* > .5) and 800 ms (*p* > .5). Similarly, a follow-up ANOVA with Target Location (typical vs. atypical) as a within-subjects factor and SOA (250 ms vs. 100 ms and 800 ms) as a between-subjects factor showed a significant interaction, *F*(2, 58) = 9.394, *p* = .003, η_p_^2^ = 0.139. Again, planned *t* tests showed shorter RTs to targets in typical versus atypical location, *t*(19) = −4.325, *p* < .001, at SOA 250 ms, whereas there was no significant difference at 100 ms and 800 ms (*t* < 1).

## General Discussion

Three experiments were conducted to investigate the nature and time course of perceptual simulations during word processing. Specifically, we tested the hypothesis that comprehending words with spatial associations involves reactivating relevant memories of typical situations by means of mental simulation. To that end, participants were asked to discriminate between two pictures either appearing at the location cued by a prime word or at the opposite location. Critically, the spatial primes either denoted entities that typically co-occur with one of the targets, or unrelated ones. Prime word duration (and, thereby, SOA) was 250 ms in Experiment 1, 100 ms in Experiment 2, and 800 ms in Experiment 3.

The first main finding was that although all three experiments found significant priming for prime words associated with the target picture, only in Experiment 1 (SOA = 250 ms) was the priming effect location specific: Targets associated with the prime words were responded to faster when appearing in the cued location compared with a statistically nonsignificant slowing down in Experiments 2 and 3. This suggests that within 250 ms (but not before 100 ms), postword onset perceptual simulations are triggered that involve a typical situation from which we know the respective object including typically co-occurring objects. In the following couple of hundreds of milliseconds, the simulation decays and the visuospatial system returns to baseline level.

The second main finding was a robust advantage for targets appearing in their typical location in Experiment 1, whereas this effect was again absent in Experiments 2 and 3. The finding that this effect was also specific to the SOA of 250 ms is further evidence for the activation of the visuospatial system in a very specific time window in which it responds to objects in locations compatible with past perceptual experience. This temporal specificity to the very time window that has been recently proposed to be the one in which semantic information is accessed (Moseley Pulvermüller, & Shtyrov, 2013; Pulvermüller, Shtyrov, & Hauk, 2009) makes simulations a likely candidate to be one of the semantic mechanisms engaged.

Our findings provide new evidence on when perceptual simulation of visually presented words occurs. For instance, Zwaan, Stanfield, and Yaxley (2002) found that, after reading a sentence about an eagle flying in the sky (as opposed to sitting on a branch), participants were faster at judging that an eagle was mentioned in the sentence when presented with a picture of an eagle with outstretched wings (as opposed to perched wings). Perceptual simulations are found in children from the age of 7 years, suggesting that they are a central mechanism that emerges when language skills (reading) are still developing (Engelen, Bouwmeester, de Bruin, & Zwaan, 2011), they are activated during memory retrieval (Hofstetter, Achaibou, & Vuilleumier, 2012) and are present in second-language processing (De Grauwe, Willems, Rueschemeyer, Lemhöfer, & Schriefers, 2014). Considerable evidence supports the notion of a flexible, modality-specific embodiment (Binder & Desai, 2011; Martin, Wiggs, Ungerleider, & Haxby, 1996; Hoenig, Sim, Bochev, Herrnberger, & Kiefer, 2008; Kiefer et al., 2012), such that concepts can selectively draw upon auditory regions in superior posterior and middle temporal gyri (Kiefer, Sim, Herrnberger, Grothe, & Hoenig, 2008), gustatory regions (Simmons, Martin, & Barsalou, 2005), primary olfactory cortex (González et al., 2006), or motor regions (Pulvermüller, Shtyrov, & Ilmoniemi, 2005).

Importantly, the present findings allow for a more precise characterization of what simulations may look like and when they occur: In line with accounts of situated cognition (Barsalou, 2009; Crocker, Knoeferle, & Mayberry, 2010; Yeh & Barsalou, 2006), they suggest that word meanings are grounded in situations in which the denoted entity is typically encountered. Here, words were presented without contextual information, and it was hypothesized that they would elicit simulations of prototypical visual events associated with them, thereby facilitating the identification of targets belonging to the respective event. On the same grounds, it was hypothesized that words with an up/down association that do not belong to the target’s prototypical event should not facilitate target identification in congruent location. These hypotheses were borne out, suggesting that concepts are not understood in isolation, but are framed against a background event within which they usually occur. This is a rather different position compared with previous proposals that congruency effects result from attentional shifts to the location implied by the cue words (e.g., Chasteen et al., 2010; Dudschig et al., 2012), even though a vertical shift of attention likely follows from event activation. Although the attentional shift account predicts congruency effects whenever the target location matches the prime word’s spatial association, the event activation approach predicts differential effects depending on the event relatedness of prime and target.

Moreover, the event hypothesis predicts the pattern that facilitation can turn into interference when SOA is short and cue words are from different categories (Gozli et al., 2013): Event simulation can interfere with the parallel identification of event-unrelated targets to the extent that they do not share features with the simulated entities. However, we did not find evidence for interference in our study. One reason might be that the target pictures had a spatial association themselves (e.g., a bird is typically up), which may have masked it at least in Experiment 1. The interference effect tends to be small even when meaningless symbols are used as targets, and it is possible that spatial bias (as a result of event activation) and interference are at work simultaneously and can cancel each other out.

We propose that event activation carried out in sensorimotor brain areas is based on previous perceptual experience with the denoted objects, including frequently co-occurring (background) items and their spatial location. The preactivation of the corresponding memory traces in the visual system makes it more sensitive for the congruency of subsequently appearing targets with their typical location. This is in stark contrast to the perceptual matching account recently proposed by Estes et al. (2015), which provides an alternative explanation intended to rule out simulation as the main mechanism in the current paradigm. Their idea is that words trigger a visual search for their referent in the associated location, which they describe as an “object code” and a “location code.” When both codes are consistent, for example, when the expected referent appears in the expected location or when an unexpected object appears in the unexpected location, fast responses are predicted to result. Conversely, when the codes are inconsistent, slower responses should result. Their main finding (using the same paradigm as in Estes et al., 2008) was that prime words’ visual strength did not modulate the size of the interference effect, whereas the strength of spatial association did, and that there was no difference between concrete and abstract words in terms of effect size. This led the authors to reject the simulation account based on the assumption that abstract word meanings have nonvisual representations, and that there is thus nothing visual to be simulated that could interfere with visual perception. However, it is unclear why abstract words would then trigger a visual search in their study, assuming that they do not have visual features. Thus, although the finding of interference triggered by abstract words is certainly an interesting phenomenon, it may not falsify a simulation account.

We observed no simulation effects at SOAs of 100 ms (Experiment 2) and 800 ms (Experiment 3). Rather, event-related prime-target pairs showed a semantic priming effect independent of target location. The finding that spatially relevant simulations are absent at 100 ms poststimulus lends support to the idea that there is another, additional route of processing based on statistical linguistic knowledge (Andrews et al., 2009; Barsalou et al., 2008). Barsalou et al. (2008) present evidence that such a linguistic system allows for shallow conceptual processing and can get active when the task does not require deep concept understanding. By this view, the absence of embodied effects at this very early stage (100 ms) suggests that the linguistic system carries out superficial processing and is followed by full conceptual processing based on sensorimotor simulations shortly after. It is important to note that the current study specifically taps the activation of representations coding spatial location and event structures.

Thus, although previous and our own findings are consistent with a functional role for perceptual simulation, there seem to be semantic effects for which perceptual simulations are not necessary. One way to further address this question is to determine to what extent simulations are automatically activated whenever words are encountered and to what extent they are task-induced. The first would indicate that they are necessary for word comprehension, whereas the second would indicate that they can be activated additionally to other mechanisms processing the core meaning of words. Note that perhaps the central issue here is to better understand what constitutes the core meaning of a word, and whether these core meanings are always activated regardless of context (Tanenhaus, Leiman, & Seidenberg, 1979), or whether context-independent core features do not exist (Evans, 2009). Along these lines, the automatic activation of embodied representations has recently been challenged by Lebois and colleagues (2015). Their account predicts congruency effects only when the vertical axis is made salient through the experimental instructions or the setup. In the experiments reported here, we avoided making the vertical axis salient by (a) not mentioning it in the instructions, and (b) presenting targets in central position additionally to up and down positions. Post hoc questionnaires confirmed that none of the participants suspected it to be of relevance for the experiment. This suggests that we observed automatic processes that could tap into core meanings (at least in the sense of default meanings that can be activated when comprehension processes are not modulated by contextual information). Further evidence for automatic sensorimotor activation comes from subliminal priming studies that have repeatedly reported congruency effects (Ansorge, Kiefer, Khalid, Grassl, & König, 2010; Boulenger et al., 2008; Dudschig, de la Vega, De Filippis, & Kaup, 2014), suggesting an automatic role of simulations. A first step toward establishing their necessity was made by Pulvermüller, Hauk, Nikulin, and Ilmoniemi (2005), who found faster RTs for arm- and leg-related words when the corresponding area in the motor cortex was stimulated with transcranial magnetic stimulation. Further investigations of this type are needed to test this effect’s robustness and extend it to other modalities.

It is important to note that investigating single-word comprehension can only be considered a first step toward an understanding of how natural language is processed. The current proposal is that word comprehension involves the simulation of a prototypical situation in which we frequently see the object in question. But this is not to say that words are processed in a static, monolithic way. Rather, the idea of framing concepts within backgrounds encourages dynamic processing that constantly implements contextual information. Not surprisingly, there is converging evidence from neuroimaging studies that sensorimotor simulations are indeed highly context dependent (Hoenig et al., 2008; Raposo, Moss, Stamatakis, & Tyler, 2009; van Dam, Brazil, Bekkering, & Rueschemeyer, 2014; van Dam, Rueschemeyer, Lindemann, & Bekkering, 2010; van Dam, Van Dongen, Bekkering, & Rueschemeyer, 2012). Combined with our findings, it appears that contextual modulations of simulated events are a promising direction for future research. Although considerable evidence from single-word studies supports the notion of a flexible, modality-specific embodiment (Binder & Desai, 2011; Hoenig et al., 2008; Kiefer et al., 2012; Martin et al., 1996), it has yet to be shown how concepts are integrated during natural language processing.

A possible limitation of the present study is related to the choice of targets associated with some of the prime words. Our interpretation of the location-specific priming effect in Experiment 1 is that, in the event congruent condition, target identification benefitted from the automatic event activation during word comprehension. However, when subjects processed primes related to one of the targets, it is possible that processing was influenced by this congruency and an event was created online to integrate the two concepts. Although possible, we do not believe that event activation was task-induced, because we should have observed location-specific priming in Experiment 3 (SOA 800 ms), in which there was more time to create it. Finding this effect at 250 ms only, when automatic semantic processing is likely to happen, makes our interpretation much more plausible than a cascading effect, as proposed by Mahon and Caramazza (2008).

## Conclusion

Three experiments suggest that word comprehension automatically triggers the simulation of a prototypical situation associated with the word. The identification of items typically co-occurring with words read by the participants was facilitated 250 ms after stimulus onset, but only when the item was presented in the vertical location where it is typically seen. These novel findings contribute to a better understanding and characterization of perceptual simulations.

## Figures and Tables

**Table 1 tbl1:** Spatial Association Ratings

Word type	Categories	Mean difference	*p* value
Up-words	1–2	.1	.626
	1–3	–1.19	.002
	2–3	–1.29	<.001
Down-words	1–2	–.4	.121
	1–3	.6	.019
	2–3	1.0	<.001
*Note.* Ratings of spatial association were broken down into a priori categories (1 = event-related; 2 = spatially congruent; 3 = Control) and Words with upward association (Up-words) and downward association (Down-words).			

**Table 2 tbl2:** Prime-Target Relatedness Ratings

Prime type	Up-words	Down-words	Neutral words
Event-related	1.3	4.5	—
Spatially related	3.1	2.8	—
Control	—	—	3.0
*Note.* Ratings of prime-target relatedness on a 5-point scale (1 = Usually co-occurs with up-target; 5 = usually co-occurs with down-target; 3 = Usually co-occurs with neither of the two) for event-related, spatially related and control items.			

## Footnotes

1 An overview of the blocks with all pictures and words we used is provided in the Appendix.

2 Although Pecher, Van Dantzig, Boot, Zanolie, and Huber (2010) argued against the PPC as an explanation of congruency effects in the present paradigm (see also Dantzig & Pecher, 2011), we considered the inclusion of centrally presented targets a sensible additional precaution, because their data do not fully rule out a polarity explanation (Lakens, 2011).

3 Note that this factor could not be included in a 2 × 2 × 2 ANOVA because it is not orthogonal to the other two factors.

## Overview of the Experimental Stimuli

**Table:**

Block 1	Block 2	Block 3	Block 4

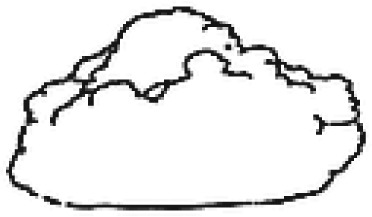
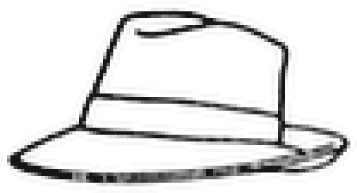
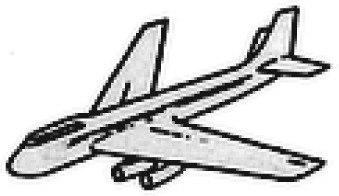
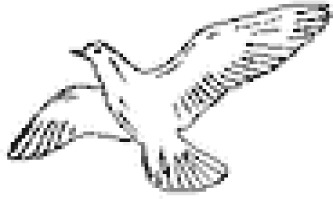
			
sky, cap	head, light	cockpit, forehead	nest, peaks
skin, beer	note, room	cocktail, parasite	taxi, wood
glass, horse	flute, brick	cake, cube	shirt, cream
foot, root	pond, toe	metro, snake	cheese, carpet

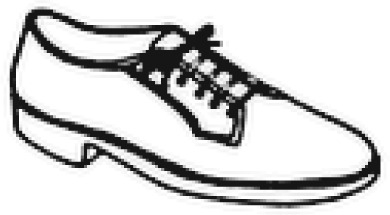
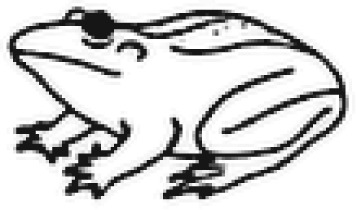
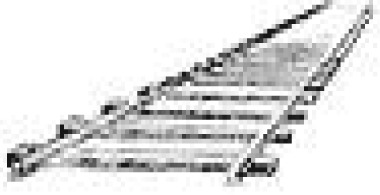
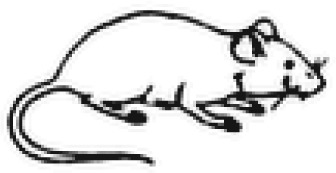
			
Block 5	Block 6	Block 7	Block 8

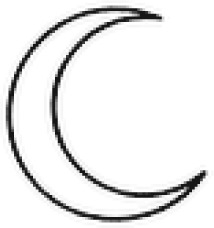
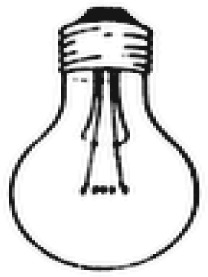
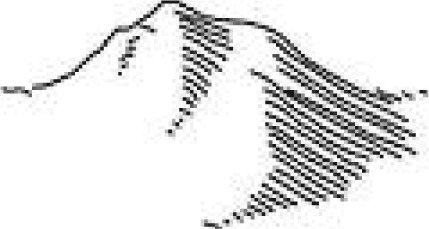
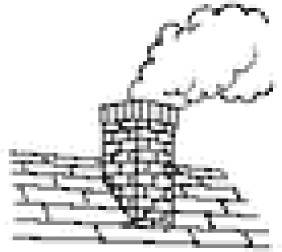
			
stars, tower	lamp, leaf	summit, rainbow	roof, caps
exit, boys	curve, knife	margin, cycles	glue, corn
wardrobe, pastry	chip, bus	juice, brush	milk, paint
blossom, asphalt	sea, floor	soil, cave	boat, bin
			
